# Selection for altruism through random drift in variable size populations

**DOI:** 10.1186/1471-2148-12-61

**Published:** 2012-05-10

**Authors:** Bahram Houchmandzadeh, Marcel Vallade

**Affiliations:** 1 Univ. Grenoble 1/CNRS, LIPhy UMR 5588, Grenoble, F-38401, France

**Keywords:** Frequency independent fitness, Genetic drif, Fixation probabilities, Non-structured populations

## Abstract

**Background:**

Altruistic behavior is defined as helping others at a cost to oneself and a lowered fitness. The lower fitness implies that altruists should be selected against, which is in contradiction with their widespread presence is nature. Present models of selection for altruism (kin or multilevel) show that altruistic behaviors can have ‘hidden’ advantages if the ‘common good’ produced by altruists is restricted to some related or unrelated groups. These models are mostly deterministic, or assume a frequency dependent fitness.

**Results:**

Evolutionary dynamics is a competition between deterministic selection pressure and stochastic events due to random sampling from one generation to the next. We show here that an altruistic allele extending the carrying capacity of the habitat can win by increasing the random drift of “selfish” alleles. In other terms, the *fixation probability* of altruistic genes can be higher than those of a selfish ones, even though altruists have a smaller fitness. Moreover when populations are geographically structured, the altruists advantage can be highly amplified and the fixation probability of selfish genes can tend toward zero. The above results are obtained both by numerical and analytical calculations. Analytical results are obtained in the limit of large populations.

**Conclusions:**

The theory we present does not involve kin or multilevel selection, but is based on the existence of random drift in variable size populations. The model is a generalization of the original Fisher-Wright and Moran models where the carrying capacity depends on the number of altruists.

## Background

Light production in *Vibrio fischeri*[1,2], siderophore production in *Pseudomonas aeruginosa*[3], invertase enzyme production in *Saccharomyces cerevisiae*[4], stalk formation by *Dictyostelium discoideum*, [2,5] are but a few examples of individuals in a community who help others at their own cost by devoting part of their resources to this task. This behavior has been termed “altruistic”. From the evolutionary point of view, altruists have a lower fitness than other individuals in the community who don’t help, but are recipient of the benefits produced by altruists. Through this paper, we will call these latter individuals ‘selfish’.

From the inception of evolution theory, the problem of the existence of altruists has been puzzling: how can a mutant with lower fitness prevail? And how does a community of altruists resist the spread of selfish allele (see [6] for a historical perspective)? In the last 40 years many models have emerged to explain the apparent contradiction between the smaller fitness of altruists and their widespread presence in various communities (for a review, see [7,8]). It is shown in these models that the actual fitness of an altruistic gene can be increased by other factors such as ‘common good’ restricted to kin (inclusive fitness [9,10]), or advantages conferred at another level of selection (group or multilevel selection [11,12]). These models which can be formulated through the Price equation have seen various generalizations and they are sometimes widely debated (see [13] and the numerous replies it has elicited).

The above models are either deterministic, *i.e.* populations change their size exactly according to their relative fitness, or involve frequency dependent fitness [14,15]. We show here that another possibility exists: an altruistic individual can produce a common good benefiting *everybody* in the community regardless of its nature (altruistic or selfish) and therefore increasing the carrying capacity of the habitat. Even though selfish individuals have always a higher fitness, genetic drift effects can favor the altruists.

It was established by the founding fathers of Population Genetics that a mutation that confers a relative fitness 1 + *s* does not automatically spread and take over the whole community, but has only a higher probability, called the *fixation probability,* to do so [16-18]. For a community of fixed size *N* of haploid individuals, the fixation probability *π* of a mutant appearing at one copy, for small selection pressure *Ns* < <1, is

(1) $$\pi \approx \frac{1}{N}+\frac{s}{2}$$

The fixation probability is composed of two terms: even in the absence of selection, the population will become homogenic; in this neutral case, all individuals at generation zero have an equal probability 1/*N* of becoming fixed. When a beneficial mutation is present, the fixation probability of its carrier is increased by the relative excess fitness.

For populations of fixed size, as can be seen from expression (1) or the more precise expression (10) obtained by Kimura [19] and Moran [20], the fixation probability is a monotonically increasing function of the sole relative fitness. In the competition between alleles, arguments based on fitness parameter alone or the fixation probability lead to the same conclusions . However, if population size is not fixed, the fixation probability *π*, which takes into account both randomness due to finite size and selection, can lead to other conclusions than the fitness parameter alone.

**Figure 1:**
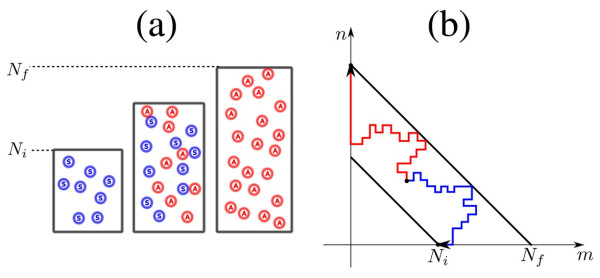
Variable carrying capacity. (**a**): A community where the carrying capacity is an increasing function of altruist number, varying from *N*_*i*_ when the population is composed only of selfish individuals to *N*_*f*_ (*N*_*f*_ > *N*_*i*_) when only altruists are present. (**b**) Two examples of random walks describing the stochastic behavior a such a system (transition probabilities 4–7), where *m,n* are the number of selfish and altruistic individuals. Red line: loss of *S*’s; Blue line: loss of *A*. A Moran process in this scheme corresponds to a random walk constrained to remain on an anti-diagonal line.

Consider an altruistic gene that by some means (production of a ‘common good’, limited grazing of natural resources, …) allows the carrying capacity to increase: if the community were composed only of altruists its population size would be *N*_*f*_; if it were composed only of selfish individuals the population size would be *N*_*i*_ (*N*_*i*_ < *N*_*f*_) (Figure 1a). The production of common good decreases the relative fitness of altruists by *s*.

Consider now the fixation probability *π*_*A*_ of one altruist mutant appearing in a community of *N*_*i*_ selfish individuals. A crude use of expression (1) shows that $\pi_{A}=\left(1/N_{i}\right)-s/2$. On the other hand, the fixation probability *π*_*s*_ of one selfish individual appearing in a community of *N*_*f*_  altruists is $\pi_{S}=\left(1/N_{f}\right)+s/2$. We see that if

(2) $$s<\frac{1}{N_{i}}-\frac{1}{N_{f}}$$

*i.e.* the cost to the altruist is smaller than the benefits in term of relative population increase, then an altruist has a larger fixation probability than a selfish one, *even though its relative fitness is smaller*. The relative advantage of a selfish mutant is compensated by the increased ‘random noise’ to which it is exposed. Note that in a deterministic model of the above process, the *A* always lose, since *S* individuals always increase their proportion.

The above argument will be refined in the following. In the next section, we formulate precisely the stochastic process of altruism outlined above by generalizing the Moran model for non-structured, well mixed populations and we show that altruists can indeed be favored in their competition with selfish individuals. We outline the amplification of this advantage in geographically structured, *viscous* populations in the third section. The final section is dedicated to concluding remarks.

## Results and discussion

### Stochastic model for altruism

The fundamental aspects of population genetics were clarified in the framework of the classical Fisher-Wright (FW) stochastic model of non-overlapping generations or its continuous time alternative introduced by Moran [20]. Moran and FW are equivalent in the limit of large populations, where both are well approximated by the same diffusion equation [21]. These are the simplest models that capture the key elements of population genetics (genetic drift, fixation probability, fixation time,…) with the fewest possible ingredients.

In the Moran model, a population of size *N* is composed of two types of individual, say *A* and *S*. Empty spots are created randomly with fixed rate *α*, increasing the carrying capacity by unity. Once an empty spot has been created, it will be colonized by the progeny of either an *A* or an *S* individual according to their proportion in the community. In order to keep the population constant, Moran added the constraint that the colonization of a new spot be followed *immediately* by the death of an individual in the community, restoring the population size to *N*. Moran is therefore a simultaneous model of duplication and annihilation; the transition probability densities for the *A* to increase or decrease their number *n* by one individual are

(3) $$W^{+}\left(n\to n+1\right)=\alpha nm\;\text{;}\;W^{-}\left(n\to n-1\right)=c\alpha nm$$

where *m* is the number of *S* individuals and *c* is the ‘cost’: 1/ *c* is the relative fitness of the *A* and *c* > 1 indicates a selective *disadvantage*. *W*^+^ stands for the probability density that the new spot is colonized by an *A* and death occurs among the *S*. In principle, a similar set of equations must be written for the *S* individuals; however, as the population size is fixed, n+m=N, the quantity *m* in eq.(3) can be replaced by *N-n* and the whole stochastic process treated as a one dimensional random walk for the *A*.

We generalize this model by including two ingredients. First, the fixed size constraint can be relaxed and we let *N* vary between two bounds *N*_*i*_ and *N*_*f*_: empty spots are created-colonized and individuals die, without these two events necessarily succeeding each other. More importantly, in order to include the effects of altruists, we suppose that the rate of creation of empty spots is proportional to the number of altruists and is equal to α*n*; in contrast, the death rate is proportional to the number of *S* individuals and is equal to α *m*. This is the simplest hypothesis that implies that the increase in the carrying capacity of the habitat is proportional to the number of altruists (see also Methods, mean field approximation).

The stochastic model that captures all these features is a two dimensional random walk with the following transition probability densities (Figure 1b):

(4) $$W\left(\left(n,m\right)\to \left(n,m+1\right)\right)=\left(N_{f}-\left(n+m\right)\right)\left(\alpha n\right)m$$

(5) $$W\left(\left(n,m\right)\to \left(n+1,m\right)\right)=\left(N_{f}-\left(n+m\right)\right)\left(\alpha n\right)n$$

(6) $$W\left(\left(n,m\right)\to \left(n,m-1\right)\right)=\left(m+n-N_{i}\right)\left(\alpha m\right)m$$

(7) $$W\left(\left(n,m\right)\to \left(n-1,m\right)\right)=c\left(m+n-N_{i}\right)\left(\alpha m\right)n$$

Consider for example the first two lines of the above equations, which are about birth events: the factor $\left(N_{f}-n-m\right)$ is the relaxation of Moran constraints and insures that population size remains below *N*_*f*_; the factor α*n* accounts for the fact that empty spot creations are proportional to the number of *A*; finally, once a birth event has occurred, the probability for it to be an *A* or an *S* is proportional to the number of the corresponding sub-populations present at this time. The last two lines, which govern population decrease, are similar: the factor $\left(m+n-N_{i}\right)$ ensures that population size remains above *N*_*i*_; the factor α*m* is the death rate (population decrease) for everybody due to the presence of selfish individuals. The cost of altruism is included in these equations: the proportion of *A* is $n/\left(m+n\right)$, but once a death event has occurred, the probability for it to be an *A* is:

(8) $$\frac{cn}{m+cn}>\frac{n}{m+n}$$

if *c* > 1. The results below don’t change significantly if the cost of altruism is included in other rates. For example, a higher probability for an *S* to reproduce, or any combination that favors *S* over *A*. Note that if the increase/decrease rates were independent of *m* and *n*, we recover the Moran model by setting $N_{f}=N_{i}+1$, in which case each birth/death is succeeded by a death/birth event (see Methods, relation to Moran model).

The above rates ensure that if *A* are lost ( *n* = 0), the population size tends toward *N*_*i*_ and if *S* are lost ( *m* = 0), it tends toward *N*_*f*_. Note that in the mean field approximation of the above process where fluctuations are neglected and the deterministic limit is taken, the *A* are always eliminated if *c* > 1 (see Methods, mean field approximation).

In finite size populations however, fluctuations play an important role. The focus of this paper is the computation of the fixation probability of the above process and the *probability* that altruists or selfish mutants take over the community. The fixation probability *π*( **k**) of a general stochastic process beginning with the initial state **k** and fixing either to **k**_**0**_ or $k_{1}$ is the solution of Kolmogorov backward equation which is a linear set of equations [22]

(9) $$\sum_{q}\;\left(\pi \left(k\right)-\pi \left(q\right)\right)W\left(k\to q\right)=0$$

(10) $$\pi \left(k_{0}\right)=0\;\text{;}\;\pi \left(k_{1}\right)=1$$

where the sum is over all the states **q** attainable from the state **k** with transition probabilities $W\left(k\to q\right)$. For one dimensional, one step processes such as Moran, **k =**  *n* and the solution of the linear system is easily obtained [22]:

(11) $$\pi \left(n\right)=\frac{1-c^{n}}{1-c^{N}}\approx \frac{e^{-N\mu s}-1}{e^{-Ns}-1}$$

where μ=n/N is the proportion of the *A*. The approximation corresponds to the Kimura solution obtained through a backward diffusion equation [19] and $c=1/\left(1+s\right)$. Expression (1) is the first order expansion of the above expression in *s*.

For the two dimensional process (4–7) where $k=\left(m,n\right)$ is the initial number of the *S* and *A*, no closed form solution can be obtained. We can however solve equation (8) numerically by standard linear solvers or else resort to a Gillespie algorithm [23] to solve the stochastic equations (4–7) directly. Both these methods are used in this paper and the analytical approximations obtained below are compared to them.

For large populations, we use the usual diffusion equation approximation of eq.(8) [19,22]. For weak selection pressure, the diffusion approximation error for the simple Moran process is O(1/N)[24]; for more general cases, the validity of the approximation has been discussed by Zhou and Qian [25]. Setting $x=m/N_{f}\text{,}\;y=n/N_{f}\text{,}\;k=N_{i}/N_{f}\text{,}$ and denoting *π*( *x, y*) the fixation probability for the initial composition ( *x, y*), the diffusion equation reads:

(12) Fx∂xπ+y∂yπ+1/2NfGx∂xx2π+y∂yy2π+c−1H−∂yπ+1/2Nf∂yy2π=0

where

**Figure 2:**
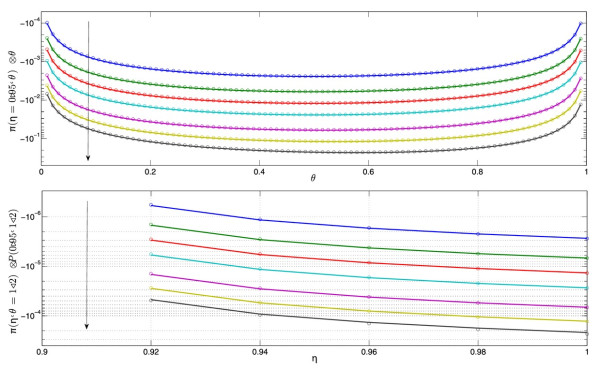
Fixation probabilities. Comparison of analytical solution (12) (solid lines) to numerical solution of eq.(8) for increasing selection pressure indicated by the arrows: $N_{f}\bar{s}=0.02,0.05,0.1,0.2,0.5,1,2$. *N*_*f*_ = 100, *N*_*i*_ = 90.

(13) F=y+kx−x+y2G=y−kx+x2−y2H=xyx+y−k

and $\pi \left(x,0\right)=0$$\pi \left(0,y\right)=1$. This is a complicated elliptic partial differential equation. In the absence of selection (*c* = 1) however, the trivial neutral solution is $\pi \left(x,y\right)=y/\left(x+y\right)$ which as expected, is just the proportion of altruists. Building upon this solution, and denoting $\mu =y/\left(x+y\right)$ for the proportion of altruists and η=x+y, we can check that to the first order of perturbation $\bar{s}=\left(c-1\right)$, the solution reads

(14) $$\pi \left(\mu ,\eta \right)=\frac{e^{N_{f}\bar{s}\mu g\left(\eta \right)}-1}{e^{N_{f}\bar{s}g\left(\eta \right)}-1}$$

where

(15) $$g\left(\eta \right)=\gamma \left(1-1/\eta N_{f}\right)$$

and *γ* is a numerical coefficient: $\gamma =1/N_{f}+\left(1+k\right)/2$. The first order perturbation solution (12), which was derived for small selection pressures $N_{f}\bar{s}\ll 1$, proves in fact to be an excellent approximation for selection pressure as high as $N_{f}\bar{s}=2$, (Figure 2).

The general solution (12) allows for the computation of the fixation probability of one individual introduced into a community of the other type. To the first order of perturbation in $\bar{s}$, the fixation probabilities *π*_*A*_ of one *A* introduced in a community of *S* reads:

(16) $$\begin{array}{c} \pi_{A}=\pi \left(m=N_{i}-1,n=1\right)\\ =\frac{1}{N_{i}}-\frac{\gamma }{2}\frac{{\left(N_{i}-1\right)}^{2}N_{f}}{N_{i}^{3}}\bar{s} \end{array}$$

and the fixation probabilities *π*_*s*_ of one *S* introduced in a community of *A* is

(17) $$\begin{array}{c} \pi_{S}=1-\pi \left(m=1,n=N_{f}-1\right)\\ =\frac{1}{N_{f}}+\frac{\gamma }{2}\frac{{\left(N_{f}-1\right)}^{2}}{N_{f}^{2}}\bar{s} \end{array}$$

**Figure 3:**
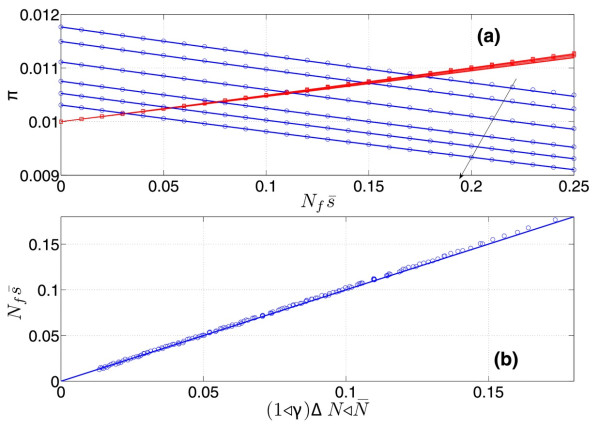
**Criterion for Altruists selection.** ( **a**) fixation probabilities *π*_*s*_ (red squares) and *π*_*A*_ (blue circles) as a function of selection pressure $N_{f}\bar{s}$, for *N*_*f*_ = 100 and $N_{i}=85,87,90,93,95,97$. Values are obtained by numerically solving eq.(8). Solid lines are theoretical values (eqs. 13,14). Increasing *N*_*i*_ are indicated by the arrow. (**b**) equilibrium selection pressure $N_{f}{\bar{s}}^{*}$ for which *π*_*A*_ = *π*_*s*_ for multiple combinations of $N_{f}\in \left[100,150\right]$ and $N_{i}\in \left[85,148\right]$, as a function of relative population increase, obtained numerically. The solid line is the theoretical value (eq.15).

Figure (3a) shows the evolution of these probabilities as a function of selection pressure for various *N*_*i*_ and *N*_*f*_. Equations (1314) show that the condition for the altruist to be favored, $\pi_{A}>\pi_{S}$, is simply

(18) $$\bar{N}\bar{s}<\bar{N}{\bar{s}}^{*}=\frac{\Delta N}{\bar{N}}$$

where $\Delta N=N_{f}-N_{i}$ and $\bar{N}=\left(N_{f}+N_{i}\right)/2$ and we have kept only the leading terms. ${\bar{s}}^{*}$ is the equilibrium relative excess cost of altruism at which *A* and *S* individuals become equivalent. Figure 3b shows the excellent agreement between the above results and exact numerical results. Altruists have a selective advantage if the *selection pressure* against them, *i.e.* the combined effect of fitness *and* population size, is smaller than the relative increase in population size. Unlike a Hamilton rule, criterion (15) is a finite size effect and is of purely stochastic nature : because of the demographic effect, selfish mutants are submitted to a higher stochastic noise than altruist; this can be sufficient to prevent them from prevailing. Note that the above computations were performed for the limiting case of weak selection (N_f_s < < 1), which is considered by most, but not all, scientists, to be the relevant limit of evolutionary dynamics [26,27]. Direct numerical resolution of eq. (8) shows however that an equilibrium excess fitness exists even at high selection pressure, given a high enough relative increase in population size.

### Geographically structured populations

**Figure 4:**
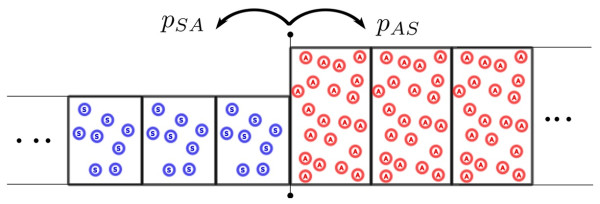
Geographically structured populations. Geographically structured population where patches can exchange migrants. For low migration rates, the border between *A* and *S* domains can be modeled as a biased random walk.

The altruists’ advantage can be enhanced for large structured populations [28-31]. Geographically structured populations can be modeled as divided into colonies that exchange migrants [32]. The Moran model on graph is a non trivial problem [33]; we restrict our treatment here to the simplest case where the migration time scale is small compared to fixation time of one mutant (viscous populations): a migrant is either lost or fixed before a new migration event happens. The argument we develop below is similar to the two level model of Traulsen and Nowak [34]. Consider a one dimensional community subdivided into *M* colonies (Figure 4), exchanging migrants with neighboring patches at rate *m*. As the migration event is rare, these colonies are fixed either into an *A* or *S* state. The probability density per unit time *p*_*SA*_ for an *S* colony on the border to become an *A* colony is to receive one migrant from the neighboring *A* colony multiplied by the probability that this mutant gets fixed:

(19) $$p_{\mathrm{SA}}=\left(mN_{f}\right)\times \pi_{A}$$

Similarly the probability density for an *A* colony on the border to become *S* is

(20) $$p_{\mathrm{AS}}=\left(mN_{i}\right)\times \pi_{S}$$

Therefore, the movement of the border itself can be considered a biased random walk. The probability Π_*A*_ for an altruist mutant to take over the whole community is thus the probability for a mutant to take over one colony and then for this colony to take over the whole community:

(21) $$\Pi_{A}=\pi_{A}\frac{1-r}{1-r^{M-1}}$$

where $r=p_{\mathrm{AS}}/p_{\mathrm{SA}}$. If the criterion (15) is satisfied, then obviously *r* < 1 and for large number of communities M > >  **1**,

(22) $$\Pi_{A}\approx \pi_{A}-\frac{N_{i}}{N_{f}}\pi_{S}>0$$

On the other hand, the probability Π_*s*_ for a selfish mutant to be fixed is

(23) $$\Pi_{S}=\pi_{S}\frac{1-\left(1/r\right)}{1-{\left(1/r\right)}^{M-1}}$$

and $\Pi_{S}\to 0$ for *M* > >  **1**: once altruists dominate, the chances for a selfish mutant to invade the community is close to zero! Increased random noise due to production of common good and a small migration rate are an efficient way of keeping selfishness in check.

The above computation concerns the low migration limit. In the high migration limit, the community is non-structured and its effective size is $\approx M\times N_{f}$. Criterion (15) shows that in this regime, altruists cannot emerge; this is indeed equivalent to the deterministic case where emergence of altruists calls for other mechanisms. Between these two regimes of high and low migration rate, there is a rich interval where migration rate is a key ingredient in the competition between altruists and cheaters.

## Conclusions

The main concepts of Population Genetics were clarified in the framework of the original model of Fisher-Wright and Moran (FWM). These models introduced the key ingredient of population size and its role in the randomness of selection. It became clear in the 1920-30’s that a beneficial mutation does not spread automatically to the whole population, but has to overcome the “random noise” of population sampling over generations. The idea that random noise plays also a role for the selection of altruism has been introduced in two kind of models, which have a marked difference with the model we present here. The first class of models, formulated mostly through evolutionary game theory formalism, concerns fixed size populations, where the transition rates are frequency dependent [14]: the fitness of an *A* individual can be superior to the fitness of an *S* individual if the number of *A* individuals already present is high enough. It can then be shown, upon very general conditions, that the fixation probability of altruists can become superior to that of selfish ones. These models can be seen as the generalization of Hamilton’s original idea, where “altruistic” help is restricted to genetically related individuals, even though Traulsen [35] has argued that the underlying mathematics is fundamentally different. The second class of models concerns group (or multilevel) selection. It has been shown [34] that the fixation probability of altruists can be higher than those of selfish ones, *if* the population is structured into groups and the splitting of one group leads to the elimination of another. It has also recently been noticed that random noise in a growing population can favor altruists during a transient period [36].

The model we present here is not frequency dependent: an *A* individual has *always* a lesser chance of reproducing than an *S* individual; the mean field description of this model has only one stable fixed point which corresponds to the disappearance of altruists. Moreover, The mechanism we propose is for non-structured populations, even though the altruist effect can be amplified when the population is structured into groups with small migration rate between groups. Imagine a group of *M* islands composed only of altruists and another group of *M* islands composed only of selfish individuals. Introduce one *S* mutant in each island of the first group and one *A* mutant in each islands of the second group. After some time, the number of islands in the first group is increased if the criterion (15) is satisfied.

In summary, we have shown, by a slight generalization of the Moran model, that in finite size populations, the fixation probability of altruists can be higher than that of selfish ones, even though their *fitness* is lower and their emergence is ‘forbidden’ by a Hamilton rule. We have also shown that in large, structured populations, and in the limit of small migration rate, the same arguments hold. Production of the ‘common good’ and increase in the carrying capacity of the habitat increase the random noise for selfish individuals and can therefore favor altruists.

The aim of the present article is not to contest the merits of kin/group selection models which have been investigated during the last forty years with a large number of case studies. We believe we are providing an alternative way of thinking about altruism which is complementary to the above models and which restores the key ingredients of population genetic to this topic.

## Methods

### Diffusion equation derivation

In the discrete backward Kolmogorov eq. (8) set $k=\left(m,n\right)$ and **q** all the states reachable from **k**, *i.e.* all states of the form ( *m* ± 1, *n*) and *m, n* ±  **1**. The equations read

(24) Wm,n→m+1,nπm,n−πm+1,n+Wm,n→m−1,nπm,n−πm−1,n+…=0

For large populations $N_{f}\gg 1$, we set $x=m/N_{f}$, $y=n/N_{f}$ and develop the above expression to the second order in $dx=dy=1/N_{f}$ (Kramers-Moyal expansion). Combining all the resulting terms leads to the partial differential equation (11). It is fruitful to express this equation in terms of total relative population η=x+y and proportion of altruists $\mu =y/\left(x+y\right)$; the inside domain shown in Figure 1 then maps into the $\left[k,1\right]\times \left[0,1\right]$ rectangle, where $k=N_{i}/N_{f}$. In these coordinates, the diffusion equation reads:

(25) Fη∂ηπ+12NfGη2∂ηηπ+μ1−μ∂μμπ−(c−1)Hη∂ηπ+1−μ∂μπ+Oc−1Nf=0

where

(26) F=ηk−η+1−kμG=−k+η1−2μ+1+kμH=ηη−kμ1−μ

### Mean field approximation

In the deterministic approximation, fluctuations are neglected. Denoting by *m* and *n* the ensemble average of the number of *S* and *A* individuals, their deterministic evolution equation reads:

(27) dmdt=Wm,n→m+1,n−Wm,n→m−1,ndndt=Wm,n→m,n+1−Wm,n→m,n−1

It is more fruitful to write directly the evolution of the proportion of *A-*individuals $\mu =n/\left(m+n\right)$. Using the expression for transition probabilities (4–7), we have

(28) $$\frac{1}{N_{f}^{2}\alpha }\frac{d\mu }{dt}=-(c-1)\eta (\eta -k)\mu {\left(1-\mu \right)}^{2}$$

where $\eta =(m+n)/N_{f}$ and $k=N_{i}/N_{f}$. It is then obvious that for c>1, dμ/dt<0. In the deterministic model, *A-*individuals always disappear.

The equation for total population reads

(29) $$\frac{1}{N_{f}^{2}\alpha }\frac{d\eta }{dt}=\eta^{2}\left(\left(1-k\right)\mu +k-\eta \right)-\left(c-1\right)\left(\eta -k\right)\mu \left(1-\mu \right)$$

for $\left(c-1\right)\ll 1$, the stationary solution of this equation, assuming that *μ* is held constant is

(30) $$\eta =k+\left(1-k\right)\mu -\left(c-1\right)\frac{\left(1-k\right)\mu^{2}\left(1-\mu \right)}{{\left(k+\left(1-k\right)\mu \right)}^{2}}$$

which shows that the increase in carrying capacity of the habitat *η - k*, at small selection pressure, is mostly proportional to the number of *A-*individuals. A closer look at the above equations (16,18) shows that η=k,μ=0 is the only stable fixed point when *c* >  **1**.

### Relation to Moran model

In a simple model where population size is variable, but birth and death rates are independent of the number of altruists and selfish individuals, a constant α will replace (α*n*) and (α *m*) in equations (4–7). In the case where $N_{f}=N_{i}+1$, the stochastic movement pictured in Figure 1b reduces to a movement on the anti-diagonal staircase: births and deaths occur only when the total population N=m+n is respectively equal to *N*_*i*_ and *N*_*f*_. The analog of the Moran process is obtained by computing the two steps transition probabilities $W^{M}\left(n,m\to n\pm 1,m\mp 1\right)$. If $m+n=N_{i}$, this implies first the birth of one individual of one type and then the death of an individual of the other type. Combining the rates given by eqs.(4–7) where birth and death rates are constant, we obtain

(31) WMn,m→n+1,m−1=α2mnWMn,m→n−1,m+1=cα2mn

The same expression is obtained if $m+n=N_{f}$.

### Numerical resolution of fixation probabilities

Two different kinds of numerical resolution were used to check the validity of our analytical results on the fixation probabilities: A Gillespie stochastic algorithm and direct resolution of eq. (8).

### Gillespie algorithm

The stochastic equations given by the rates (4–7) can be seen as 2 chemical reactions for the species $A_{i}=\left\{A,S\right\}$:

(32) $$A_{i}\overset{k_{i}^{+}}{\to }2A_{i}\;\text{;}\;A_{i}\overset{k_{i}^{-}}{\to }Ø$$

which we solve by the classical Gillespie algorithm [23] written in C++. We are interested here only in the fixation probability and not in the fixation time; the program can therefore be accelerated by computing only the nature of the event that occurs at each turn (and not its time of occurrence). In general, to solve for the fixation probability, *R* = 10^6^ stochastic trajectories are generated.

### Direct resolution

**Figure 5:**
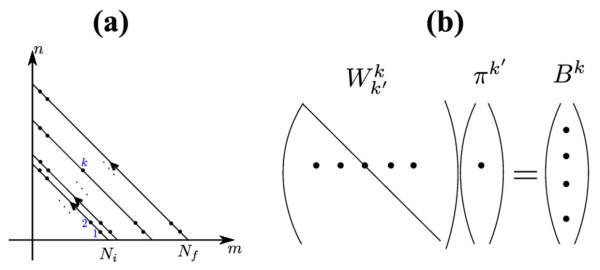
Tensor reindexation. (**a**) To each 2 d index ( *m,n*), a new 1 d index *k* is associated by scanning sequentially the anti-diagonal lines. ( **b**) The re-indexation transforms the tensorial equation (8) into a normal linear system $W_{k^{\prime }}^{k}\pi^{k^{\prime }}=B^{k}$, where *π*^*k*^ are the unknowns.

Equation (8) constitutes a linear system and can therefore be solved by standard numerical packages. For the present case however, the unknowns, *i.e.* the fixation probabilities *π*( *m,n*) don’t constitute a vector but a second rank tensor; the tensor formed from the rates *W* is of rank 4. To adapt our linear system to standard linear solvers, we have to re-index the unknowns and decrease their rank by one: $\left(m,n\right)\mapsto k$. We have chosen the following scheme, which corresponds to a sequential scanning of the anti-diagonal lines (Figure 5a):

(33) $$k\left(m,n\right)=\delta \left(N_{i}-1+\left(\delta -1\right)/2\right)+n$$

where $\delta =m+n-N_{i}$. The $\left(m,n\right)$ points belong to the interior of the trapezoid $N_{i}\leq m+n\leq N_{f}$, *n* ≥  **1**, *m* ≥  **1**.

The re-indexation transforms the equation (8) into a normal linear system

(34) $$\sum_{k^{\prime }\in I(k)}\;\left(\pi \left(k^{\prime }\right)-\pi \left(k\right)\right)W\left(k\to k^{\prime }\right)=0$$

where *I(k)* designates the 1 d indexes of the four nearest neighbors of the point ( *m,n*), where $k=k\left(m,n\right)$. The above equations can be written in standard matrix notation

(35) $$W_{k^{\prime }}^{k}\pi^{k^{\prime }}=B^{k}$$

where $\pi^{k^{\prime }}$ are the unknowns. $W_{k^{\prime }}^{k}$ is a sparse matrix, which apart from the diagonal elements, has at most four non-zero elements per line: if *k* is the image of element ( *m,n*), then $W_{k^{\prime }}^{k}\neq 0$ only if *k*′ is the image of one of the four nearest neighbors of ( *m,n*), in which case its value is given according to rates (4–7). The right hand side vector *B*^*k*^ is a sparse vector provided by the limit conditions π(m=0,n)=1: if *k*′, one of the 4 nearest neighbors of the element *k* belongs to the border *m* = 0, then $\pi^{k^{\prime }}=1$ and the corresponding $W_{k^{\prime }}^{k}$ is transferred to the right hand side to constitute the vector *B*^*k*^. Note that because we index the interior of the trapezoid, the index *k* itself can never belong to the border.

Once the linear system (20) has been constituted, it can be solved by any linear solver. We have used the commercial package matlab for these manipulations.

## Authors’ contributions

BH designed the reasearch, performed the numerical work and wrote the article. BH and MV performed the analytical computations. Both authors read and approved the final manuscript.
